# Supplementary figures and images for: Structural models for the design of novel antiviral agents against Greek Goat Encephalitis
Source: PeerJ. 2014 Nov 6;2:e664. doi: 10.7717/peerj.664 (PMC4226726; doi:10.7717/peerj.664)

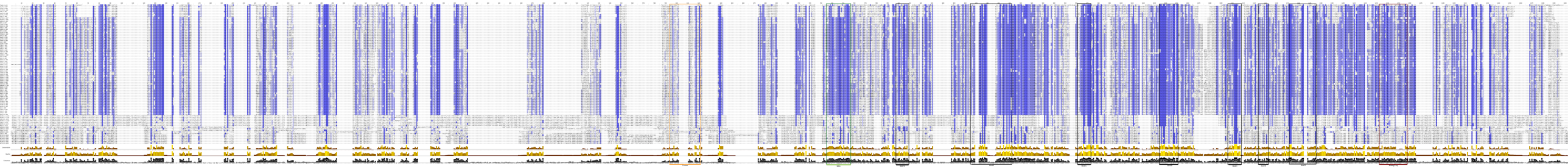

Supplement: Figure S2 — The alignment was generated using Muscle, and visualized with Jalview. Amino acids are colored blue based on percent identity in the alignment, the consensus weblogo is shown at the bottom. [file peerj-02-664-s002.png]
