# Supplementary material for: Structural models for the design of novel antiviral agents against Greek Goat Encephalitis
Source: PeerJ. 2014 Nov 6;2:e664. doi: 10.7717/peerj.664 (PMC4226726; doi:10.7717/peerj.664)
Supplement: Table S2 [file peerj-02-664-s006.docx]

**Supplementary Table 2:** **Model description and data validation.**

| **Model** | **Motifs** | **Template** | **Resolution** Å | **Reference** | **Identity %** | **Similarity %** |
| --- | --- | --- | --- | --- | --- | --- |
| NS3 Helicase | 7 | 1YKS | 1.8 | [Wu, et al., 2005](#_ENREF_35) | 50 | 65 |
| NS5 RdRP | 8 | 4K6M | 2.6 | [Lu and Gong, 2013](#_ENREF_15) | 58 | 73 |
