# Supplementary material for: Structural models for the design of novel antiviral agents against Greek Goat Encephalitis
Source: PeerJ. 2014 Nov 6;2:e664. doi: 10.7717/peerj.664 (PMC4226726; doi:10.7717/peerj.664)
Supplement: Figure S1 — The different complexes that are thought to arise in different cellular compartments during and following polyprotein processing are shown underneath. [file peerj-02-664-s001.pdf]

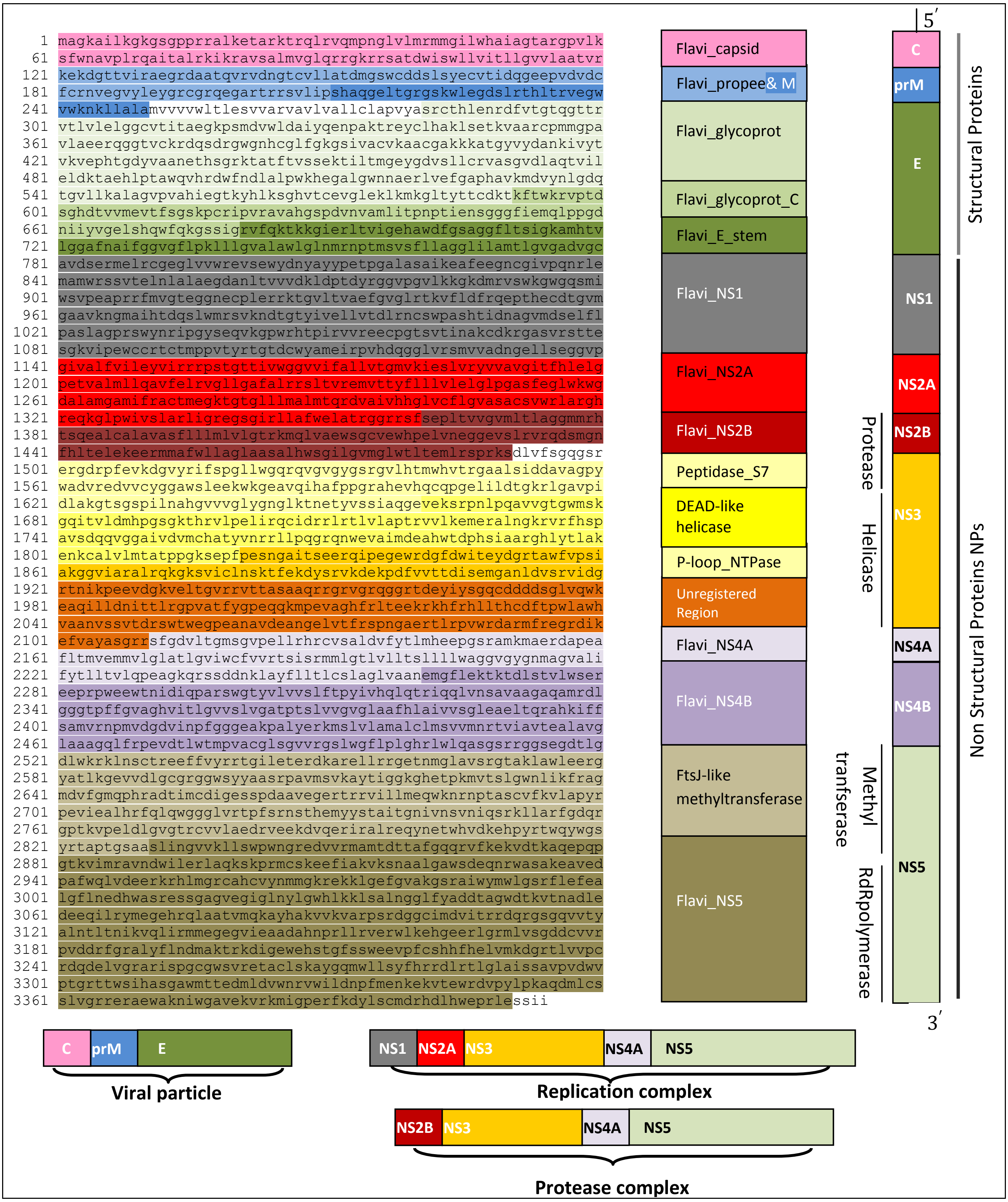

1 magkailkgkgsgpprralketarktrqlrvqmpnglvlmrmmgilwhaiagtargpvlk
61 sfwnavplrqaitalrkikravsalmvglqrrgkrrsatdwiswllvitllgvvlaatvr
121 kekdgttviraegrdaatqvrvdngtcvllatdmgswcddslsyecvtidqgeepvdvdc
181 fcrnvegvyleygrcgrqegartrrsvlipshaqgeltgrgskwlegdslrthltrvegw
241 vwknkllalamvvvvwltlesvvarvavlvallclapvyasrcthlenrdfvtgtqgttr
301 vtlvlelggcvtitaegkpsmdvwldaiyqenpaktreyclhaklsetkvaarcpmmgpa
361 vlaeerqggtvckrdqsdrgwgnhcglfgkgsivacvkaacgakkkatgyvydankivyt
421 vkvephtgdyvaanethsgrktatftvssektiltmgeygdvsllcrvasgvdlaqtvil
481 eldktaehlptawqvhrdwfndlalpwkhegalgwnnaerlvefgaphavkmdvynlgdq
541 tgvllkalagvpvahiegtkyhlksghvtcevglekl kmkgltyttcdktkftwkrvptd
601 sghdtvvmevtfsgskpcripvravahgspdvnvamlitpnptiensgggfiemqlppgd
661 niiyvgelshqwfqkgssigrvfqktkkgierltvigehawdfgsaggfltsigkamhtv
721 lggafnaifggvgflpklllgvalawlglnmrnptmsvsfllagglilamtlgvgadvgc
781 avdsermelrcgeglvvwrevsewydnyayypetpgalasaikeafeegncgivpqnrle
841 mamwrssvtelnlalaegdanltvvvdkldptdyrggvpgvlkkgkdmrvswkgwgqsmi
901 wsvpeaprrfmvgteggnecplerrktgvltvaefgvglrtkvfldfrqepthecdtgvm
961 gaavkngmaihtdqslwmrsvkndtgtyivellvtdlrncswpashtidnagvmdselfl
1021 paslagprswynripgyseqvkgpwrhtpirvvreecpgtsvtinakcdkrgasvrstte
1081 sgkvipewccrtctmppvtyrtgtdcwyameirpvhdqgglvrsmvvadngellseggvp
1141 givalfvileyvirrrpstgttivwggvvifallvtgmvkieslvryvvavgitfhlelg
1201 petvalmllqavfelrvgllgafalrrsltvremvttyflllvlelglpgasfeglwkwg
1261 dalamgamifractmegktgtglllmalmtqrdvaivhhglvcflgvasacsvwrlargh
1321 reqkglpwivslarligregsgirllafwelatrggrrsfsepltvvgvmltlaggmmrh
1381 tsqealcalavasflllmlvlgtrkmqlvaewsgcvewhpelvneggevslrvrqdsmgn
1441 fhltelekeermmafwllaglaasalhwsgilgvmglwtltemlrsprksdlvfsgqgsr
1501 ergdrpfevkdgvyrifspgllwgqrqvgvgygsrgvlhtmwhvtrgaalsiddavagpy
1561 wadvredvvcyggawsleekwkgeavqihafppgrahevhqcqpgelildtgkrlgavpi
1621 dlakgtsgspilnahgvvvglygnglktnetyvssiaqgeveksrpnlpqavvgtgwmsk
1681 gqitvldmhpgsgkthrvlpelirqcidrrlrtlvlaptrvvlkemeralngkrvrfhsp
1741 avsdqqvggaivdvmchatyvnrrllpqgrqnwevaimdeahwtdphsiaarghlytlak
1801 enkcalvlmtatppgksepfpesngaitseerqipegewrdgfdwiteydgrtawfvpsi
1861 akggviaralrqkgksviclnsktfekdysrvkdekpdfvvttdisemganldvsrvidg
1921 rtnikpeevdgkveltgvrrvttasaaqrrgrvgrqggrtdeyiysgqcddddsglvqwk
1981 eaqilldnittlrgpvatfygpeqqkmpevaghfrlteekrkhfrhllthcdftpwlawh
2041 vaanvssvtdrswtwegpeanavdeangelvtfrspngaertlrpvwrdarmfregrdik
2101 efvayasgrrsfgdvltgmsgvpellrhrcvsaldvfytlmheepgsramkmaerdapea
2161 fltmvemmvlglatlgviwcfvvrtsisrmmlgtlvlltsllllwaggvgygnmagvali
2221 fytlltvlqpeagkqrssddnklayflltlcslaglvaanemgflektktdlstvlwser
2281 eeprpweewtnidiqparswgtyvlvvslftpyivhqlqtriqqlvnsavaagaqamrdl
2341 gggtpffgvaghvitlgvvslvgatptslvvgvglaafhlaivvsgleaeltqrahkiff
2401 samvrnpmvdgdvinpfgggeakpalyerkmslvlamalclmsvvmnrtviavtealavg
2461 laaagqlfrpevdtlwtmpvacglsgvvrgslwgflplghrlwlqasgsrrggsegdtlg
2521 dlwkrklnsctreeffvyrrtgileterdkarellrrgetnmglavsrgtaklawleerg
2581 yatlkgevvdlgcgrggwsyyaasrpavmsvkaytiggkghetpkmvtslgwnlikfrag
2641 mdvfgmqphradtimcdigesspdaavegertrrvillmeqwknrnptascvfkvlapyr
2701 peviealhrfqlqwggglvrtpfsrnsthemyystaitgnivnsvniqsrkllarfgdqr
2761 gptkvpeldlgvgtrcvvlaedrveekdvqeriralreqynetwhvdkehpyrtwqywgs
2821 yrtaptgsaaslingvvkllswpwngredvvrmamtdttafgqqrvfkekvdtkaqepqp
2881 gtkvimravndwilerlaqkskprmcskeefiakvksnaalgawsdeqnrwasakeaved
2941 pafwqlvdeerkrhlmgrcahcvynmmgkrekklgefgvakgsraiwymwlgsrflefea
3001 lgflnedhwasressgagvegiglnylgwhlkklsalngglfyaddtagwdtkvtnadle
3061 deeqilrymegehrqlaatvmqkayhakvvkvarpsrdggcimdvitrrdqrgsgqvvty
3121 alntltnikvqlirmmegegvieaadahnprllrverwlkehgeerlgrmlvsgddcvvr
3181 pvddrfgralyflndmaktrkdigewehstgfssweevpfcshhfhelvmkdgrtlvvpc
3241 rdqdelvgrarispgcgwsvretaclskaygqmwllsyfhrrdlrtlglaissavpvdwv
3301 ptgrttwsihasgawmttedmldvwnrvwildnpfmenkekvtewrdvpylpkaqdmlcs
3361 slvgrreraewakniwgavekvrkmigperfkdylscmdrhdlhwepriessii
Flavi_capsid
Flavi_propee& M
Flavi_glycoprot
Flavi_glycoprot_C
Flavi_E_stem
Flavi_NS1
Flavi_NS2A
Flavi_NS2B
Peptidase_S7
DEAD-like helicase
P-loop_NTPase
Unregistered Region
Flavi_NS4A
Flavi_NS4B
FtsJ-like methyltransferase
Flavi_NS5
5′
C
prM
E
NS1
NS2A
NS2B
NS3
NS4A
NS4B
NS5
3′
Structural Proteins
Non Structural Proteins NPs
Protease
Helicase
Methyl tranfserase
RdRpolymerase
C
prM
E
Viral particle
NS1
NS2A
NS3
NS4A
NS5
Replication complex
NS2B
NS3
NS4A
NS5
Protease complex
